# When Sight and Cancer Collide: A Rare Case of Paraneoplastic Bilateral Optic Neuritis

**DOI:** 10.7759/cureus.49923

**Published:** 2023-12-04

**Authors:** Jorge Nadal Bosch, Mario Moya, Samuel Serna, Roberto A Cruz, Javier Malcolm

**Affiliations:** 1 Diagnostic Radiology, Doctors Hospital at Renaissance, Edinburg, USA; 2 Radiology, Doctors Hospital at Renaissance, Edinburg, USA; 3 Neurology, Doctors Hospital at Renaissance, Edinburg, USA; 4 Medical Information, Doctors Hospital at Renaissance, Edinburg, USA

**Keywords:** intravenous immunoglobulins (ivig), double negative optic neuritis, therapeutic plasmapheresis, cancer cervical, paraneoplastic optic neuropathy

## Abstract

Bilateral acute optic neuritis is a rare and challenging clinical presentation, often associated with conditions like multiple sclerosis or neuromyelitis optica spectrum disorder. We present the case of a 40-year-old woman with a complex medical history, including poorly differentiated squamous cell carcinoma of the cervix (stage IIIC), who presented with a swift and profound bilateral vision loss. Despite initial treatment with high-dose methylprednisolone and therapeutic plasma exchange, her optic nerve enhancement on MRI and negative autoantibody results raised suspicion of paraneoplastic optic neuritis. This prompted consultation with oncology, and the patient initiated chemotherapy. The rapid onset and progression of bilateral optic neuritis in the context of cervical carcinoma emphasize the importance of considering paraneoplastic syndromes in such cases. A multidisciplinary approach involving neurology, ophthalmology, and oncology specialists is vital for the diagnosis and management of these complex presentations. This case underscores the need for heightened awareness of paraneoplastic etiologies in patients with malignancies and unexplained neurological symptoms.

## Introduction

Bilateral acute optic neuritis is a rare and diagnostically challenging clinical presentation, particularly when occurring in the context of a complex medical history. We present the case of a 40-year-old female patient who, in addition to a background of hypertension and hyperlipidemia, had recently been diagnosed with poorly differentiated squamous cell carcinoma of the cervix (stage IIIC). This patient's clinical course was marked by a remarkably swift onset of bilateral vision impairment, initially characterized by blurry vision and reduced color perception, which rapidly progressed to complete loss of light perception within just three days. The abruptness of this vision loss, coupled with the patient's recent cancer diagnosis, prompted a comprehensive evaluation to explore the potential association between her visual symptoms and underlying medical conditions. Neuromyelitis optica spectrum disorder (NMOSD) is an autoimmune disorder primarily affecting the central nervous system, with a predilection for the optic nerves and spinal cord. It is characterized by recurrent episodes of optic neuritis and transverse myelitis, leading to visual and motor dysfunction. NMOSD is often associated with the presence of aquaporin-4 antibodies (AQP4-IgG), which target the aquaporin-4 water channel protein in astrocytes, contributing to inflammation and demyelination in affected regions of the nervous system [1]. This condition is known for its clinical heterogeneity and can present with a diverse range of neurological symptoms, making early diagnosis and intervention crucial [2]. Poorly differentiated squamous cell carcinoma of the cervix represents an advanced stage of cervical cancer characterized by aggressive tumor growth and the potential for distant metastasis. Its management often involves a combination of surgery, chemotherapy, and radiation therapy, with the stage of the disease influencing treatment decisions [3]. The diagnosis of cervical cancer frequently prompts a multidisciplinary approach involving gynecologic oncologists, radiation oncologists, and medical oncologists to tailor the treatment plan to the patient's specific circumstances [4]. Paraneoplastic syndromes refer to a group of rare, remote effects of cancer on distant organs or tissues, often resulting from an immune response to tumor-associated antigens. These syndromes can manifest with a wide range of neurological, dermatological, hematological, or endocrine symptoms, among others [5]. The association between cancer, particularly gynecological malignancies, and paraneoplastic syndromes is recognized, and their identification can significantly impact patient management [6].

## Case presentation

We present the case of a 40-year-old female with a medical history notable for hypertension, hyperlipidemia, and a recent diagnosis of poorly differentiated squamous cell carcinoma of the cervix, stage IIIC, made four weeks ago. The cervical cancer was complicated by obstructive bilateral hydronephrosis, which required bilateral nephrostomy tube placement in a prior medical facility. She presented to our facility with a sudden, painless bilateral loss of vision. Initially, her visual symptoms began with blurry vision, and reduced color perception, and progressed to complete loss of light perception within three days of her initial presentation. Upon arrival, her vital signs were within normal limits, with a temperature of 98.1°F, heart rate of 72, respiratory rate of 18, blood pressure of 101/60 mmHg, and oxygen saturation of 100% on room air.

On neurological examination, the patient demonstrated full alertness and orientation to person, time, and place. Her thought processes appeared normal and organized, and her speech was intact with no evidence of aphasia or dysarthria. Cranial nerve examination revealed a full range of extraocular muscle movements, absence of nystagmus, and intact facial sensation in the distributions of V1, V2, and V3 bilaterally. Facial movements were symmetric in both the upper and lower face, and the shoulder shrug was symmetric. Tongue protrusion was midline. Motor strength was 5/5 in all upper and lower extremities bilaterally, with no evidence of pronator drift. Sensory examination demonstrated intact responses to light touch in all four extremities, and coordination was preserved during finger-to-nose testing.

Ophthalmologic examination revealed normal conjunctiva and sclera. However, pupillary light reactions were absent bilaterally and the assessment of visual acuity revealed a lack of light perception, indicating a complete absence of visual responsiveness. Furthermore, the evaluation of the visual field was precluded due to the patient's profound absence of vision. Fundoscopy showed circumferential blurring of the optic disc, consistent with mild swelling, and intact vessels. Notably, the patient had nephrostomy tubes in place bilaterally, and the rest of the physical examination was unremarkable.

Laboratory studies are shown below in Table 1.

**Table 1:** Laboratory test results

Laboratory Test	Actual Result	Normal Range
White Blood Cell Count (WBC)	8.70 th/uL	4.0 - 11.0 th/uL
Hemoglobin	8.2 g/dL	12.0 - 15.5 g/dL
Hematocrit	26.10%	36.0% - 46.0%
MCV (Mean Corpuscular Volume)	76.5 fL	80.0 - 100.0 fL
Sodium	136 mmol/L	135 - 145 mmol/L
Potassium	3.6 mmol/L	3.5 - 5.0 mmol/L
Chloride	101 mmol/L	98 - 107 mmol/L
Carbon Dioxide (CO_2_)	27 mmol/L	23 - 29 mmol/L
Creatinine	0.5 mg/dL	0.6 - 1.3 mg/dL
Blood Urea Nitrogen (BUN)	16 mg/dL	7 - 20 mg/dL
Calcium	8.4 mg/dL	8.5 - 10.5 mg/dL
Glucose	127 mg/dL	70 - 99 mg/dL
AST (Aspartate Transaminase)	15 IU/L	10 - 40 IU/L
ALT (Alanine Transaminase)	18 IU/L	7 - 56 IU/L
Alkaline Phosphatase	116 IU/L	44 - 147 IU/L
Albumin	3.6 g/dL	3.4 - 5.4 g/dL
Total Bilirubin	0.3 mg/dL	0.2 - 1.2 mg/dL
Prothrombin Time (PT)	13 seconds	11 - 13 seconds
INR (International Normalized Ratio)	1.1	0.9 - 1.2
Partial Thromboplastin Time (PTT)	24.8 seconds	25 - 35 seconds
Lactic Acid	0.49	0.5 - 2.2 mmol/L
Blood Culture	Negative	Negative
Urine Culture	Negative	Negative

Magnetic resonance imaging (MRI) of the brain with and without contrast demonstrated minimal deep white matter disease within the cerebral hemispheres, suggestive of chronic small-vessel ischemic changes. No acute intracranial abnormalities or intracranial metastases were identified. A lumbar puncture was performed and results of the cerebrospinal fluid are shown in Table 2.

**Table 2:** Cerebrospinal fluid analysis

CSF Parameter	Result	Normal Values
CSF Pressure	9 mm Hg	8-15 mm Hg
CSF Appearance	Clear	Clear
CSF Protein Level	23 mg/dL	15-45 mg/dL
CSF Glucose Level	60 mg/dL	40-70 mg/dL
CSF Red Blood Cell Count	0	0
CSF White Blood Cell Count	4	0-5 cells/μL
Neutrophils (Percentage)	6%	0-6%
Lymphocytes (Percentage)	56%	40-80%
Presence of Atypical Cells	Negative	Negative
CSF Cultures	Negative	Negative

**Figure 1:**
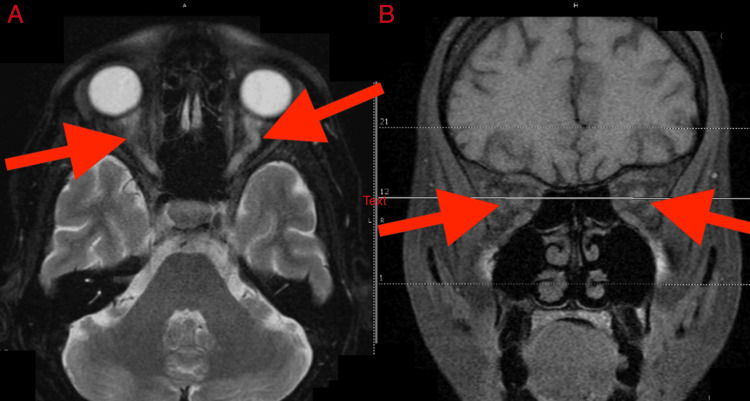
Image A presents an MRI brain T2 sequence axial section showing bilateral uniform symmetric hyperintensity in its entire length with associated optic nerve swelling and tortuosity; Image B presents an MRI brain coronal section pre-contrast showing the optic nerve swelling These distinctive features play a crucial role in diagnosing various optic nerve disorders, such as optic neuritis, optic nerve head edema, or optic nerve sheath meningiomas, which may manifest with increased T2 signal intensity and alterations in the optic nerve's anatomical characteristics.

MRI of the orbits with and without contrast demonstrated symmetric enhancement throughout the bilateral optic nerves, extending from the retrobulbar region to the prechiasmatic region, consistent with acute bilateral optic neuritis (Figure 1).

Neurology was consulted, and the patient underwent a course of five sessions of therapeutic plasma exchange (TPE) in addition to a five-day course of intravenous methylprednisolone 1 gram daily, which resulted in minimal improvement in her vision. Testing for AQP4-IgG and anti-myelin oligodendrocyte glycoprotein antibodies yielded negative results. Due to the high likelihood of paraneoplastic bilateral optic neuritis, oncology consultation was sought. The patient initiated induction chemotherapy with carboplatin and paclitaxel, completing one cycle during her hospital stay. She experienced partial improvement in vision, with the ability to perceive shadows and count fingers with both eyes. Additionally, there was a gradual improvement in pupillary light reactions bilaterally. The patient was discharged, with plans to continue chemotherapy in the outpatient setting.

Following a three-week interval, the patient exhibited partial improvement in vision, demonstrating the ability to perceive shadows and count fingers with both eyes. Moreover, there was a gradual amelioration in pupillary light reactions bilaterally. These encouraging developments were instrumental in the decision to discharge the patient with a continued plan for chemotherapy in the outpatient setting.

## Discussion

The patient's clinical presentation of bilateral acute optic neuritis, in the context of her underlying malignancy and rapid vision deterioration, presents a unique and diagnostically challenging scenario. Bilateral optic neuritis is typically associated with conditions like multiple sclerosis (MS), NMOSD, or infectious etiologies [7]. However, the association of optic neuritis with an underlying malignancy, such as cervical carcinoma, is uncommon [8].

In this particular case, the patient initially exhibited a restricted response to high-dose methylprednisolone therapy, prompting consideration of TPE. TPE, employed in instances of inadequate steroid responsiveness, involves the removal of plasma containing deleterious antibodies or toxins, subsequently replaced with donor plasma or an appropriate substitute [9]. Despite these interventions, the patient demonstrated only marginal visual amelioration. Persistent symptoms, coupled with optic nerve enhancement evident on MRI and the absence of AQP4-IgG and anti-myelin oligodendrocyte glycoprotein antibodies, raised suspicion of paraneoplastic bilateral optic neuritis [10,11].

In demyelinating disorders, such as optic neuritis, the observed enhancement on MRI is dynamic and frequently associated with the acute inflammatory phase. This enhancement reflects augmented blood-brain barrier permeability and the subsequent influx of contrast material into the afflicted region. Notably, in the context of optic neuritis and analogous demyelinating conditions, this enhancement is commonly discernible in the optic nerves.

As the inflammatory process subsides and the acute phase resolves, enhancement on MRI may decrease or disappear. This reduction in enhancement does not necessarily correlate directly with the degree of functional recovery or improvement in symptoms. In some cases, individuals may experience partial or complete recovery of visual function despite persistent or residual MRI abnormalities.

Recovery from optic neuritis and other demyelinating events can involve remyelination of damaged nerve fibers and a degree of functional compensation by the nervous system. However, the relationship between radiological findings and clinical outcomes can be complex, and not all patients will experience full normalization of MRI abnormalities, even with clinical improvement.

Radiological investigations, particularly magnetic resonance imaging MRI, played a crucial role in this case. The persistent optic nerve enhancement observed on MRI is a significant finding. Contrast-enhanced MRI is essential for assessing optic nerve involvement and can help differentiate between demyelinating diseases like multiple sclerosis and NMOSD and other potential causes such as paraneoplastic syndromes. In cases of paraneoplastic syndromes, radiological findings often reveal specific patterns of involvement, helping to guide the diagnostic process.

Differentiating between demyelinating diseases like optic neuritis, MS, NMOSD, and other potential causes, such as paraneoplastic syndromes, using MRI involves careful evaluation of imaging findings and consideration of specific patterns and characteristics associated with each condition. Table 2 provides an overview of how MRI findings can help differentiate between various neurological conditions, including MS, NMOSD, and paraneoplastic syndromes, based on lesion location, optic nerve involvement, spinal cord lesions, pattern of enhancement, clinical correlation, and consideration of underlying malignancy.

**Table 3:** MRI findings and differentiation points between optic neuritis, neuromyelitis optica spectrum disorder (NMOSD), and paraneoplastic syndrome It's important to note that while MRI findings can be helpful in distinguishing between these conditions, a comprehensive diagnosis also relies on clinical history, physical examination, cerebrospinal fluid analysis, and sometimes additional tests like blood work or antibody testing like in the case of NMOSD. A neurologist or specialist is typically responsible for interpreting these findings and making a definitive diagnosis.

Condition	MRI Findings	Key Differentiators
Optic Neuritis	- Increased T2 signal intensity in the optic nerve.	- Often unilateral involvement of the optic nerve.
	- Contrast enhancement of the affected optic nerve.	- Maybe a first clinical manifestation of MS.
	- May have periventricular white matter lesions.	
NMOSD	- Longitudinally extensive transverse myelitis (LETM).	- Extensive spinal cord involvement (LETM).
	- Extensive and longitudinally extensive optic nerve lesions.	- May involve the brain.
Paraneoplastic Syndrome	- Variable MRI findings; lesions in the brain or spinal cord.	- Associated with an underlying malignancy.
	- Lesions may mimic demyelination.	- Diagnosis requires investigation of malignancies.
Multiple Sclerosis	- Multiple, discrete lesions in the brain and spinal cord.	- Lesions often located in periventricular areas.
	- Lesions show dissemination in space and time (McDonald criteria).	- Characteristic features include Dawson's fingers.
	- May exhibit "black holes" on T1-weighted images.	- Presence of oligoclonal bands in cerebrospinal fluid.

It's important to note that while MRI findings can provide valuable information, the diagnosis of these conditions often involves a combination of clinical, radiological, and laboratory assessments. Therefore, the interpretation of MRI results should be made in conjunction with the patient's medical history, clinical presentation, and specialized antibody testing, if available, to arrive at an accurate diagnosis and guide treatment decisions.

This radiological evidence, coupled with the clinical presentation and laboratory findings, contributed to the suspicion of a paraneoplastic etiology. It prompted consultation with oncology, and the patient initiated induction chemotherapy with carboplatin and paclitaxel while hospitalized. The rapid onset and progression of bilateral optic neuritis in the setting of cervical carcinoma underscore the importance of considering paraneoplastic syndromes as potential underlying causes. Other causes of optic neuritis are shown in Table 4.

**Table 4:** Possible causes of optic neuritis

Possible Causes of Bilateral Optic Neuritis	Description
Multiple Sclerosis (MS)	MS is a chronic autoimmune disease affecting the central nervous system, including optic nerves. Bilateral optic neuritis is frequently associated with MS.
Neuromyelitis Optica (NMO)	Also known as Devic's disease, NMO is an autoimmune disorder primarily impacting optic nerves and the spinal cord. Bilateral optic neuritis can manifest as a feature of NMO.
Infections	Certain infections, both viral (e.g., herpes) and bacterial (e.g., syphilis), may lead to bilateral optic neuritis.
Autoimmune Disorders	Beyond MS and NMO, autoimmune diseases like lupus or Sjögren's syndrome can induce inflammation of the optic nerves.
Toxic or Medication-Induced	Exposure to toxins or certain medications, such as those used in tuberculosis or rheumatoid arthritis treatment, may contribute to optic neuritis.
Sarcoidosis	An inflammatory disease affecting various organs, including the eyes, sarcoidosis may lead to bilateral optic neuritis.
Connective Tissue Disorders	Conditions like systemic lupus erythematosus (SLE) or rheumatoid arthritis may involve the optic nerves and lead to bilateral optic neuritis.
Metabolic Disorders	Certain metabolic conditions, including diabetes, may contribute to the development of optic neuritis.
Vascular Disorders	Disorders affecting blood vessels, such as vasculitis, can lead to inflammation of the optic nerves, resulting in bilateral optic neuritis.
Idiopathic	In some cases, the cause of bilateral optic neuritis remains unknown (idiopathic), emphasizing the need for further investigation to discern the underlying factor.

In complex cases like this, where clinical symptoms overlap with various conditions, radiology serves as a critical diagnostic tool. It aids in narrowing down the differential diagnosis, guiding treatment decisions, and monitoring disease progression. This case highlights the pivotal role of radiology in multidisciplinary approaches to complex presentations, involving neurology, ophthalmology, and oncology specialists. Collaboration between these specialties, supported by radiological evidence, is essential to address both the malignancy and the associated neurological complications effectively.

## Conclusions

In conclusion, we present a rare and intriguing case of a 40-year-old female with a documented history of cervical carcinoma who presented with a sudden onset of bilateral optic neuritis. This distinctive clinical presentation underscores the imperative need to consider paraneoplastic bilateral optic neuritis in individuals with concurrent malignancies and unexplained neurological symptoms. The association between malignancy and paraneoplastic neurological syndromes, though infrequent, necessitates heightened clinical suspicion for prompt diagnosis. This case report contributes to the existing medical literature by shedding light on the diagnostic challenges and therapeutic complexities inherent in paraneoplastic bilateral optic neuritis. Continued research and shared clinical experiences are vital for advancing our understanding of these rare entities and refining the approach to their diagnosis and management.
